# The Relation between Nonverbal IQ and Postoperative CI Outcomes in Cochlear Implant Users: Preliminary Result

**DOI:** 10.1155/2015/313274

**Published:** 2015-07-05

**Authors:** Mina Park, Jae-Jin Song, Seo Jin Oh, Min-Sup Shin, Jun Ho Lee, Seung Ha Oh

**Affiliations:** ^1^Department of Otorhinolaryngology-Head and Neck Surgery, Seoul National University College of Medicine, Seoul National University Hospital, Seoul, Republic of Korea; ^2^Department of Otorhinolaryngology-Head and Neck Surgery, Seoul National University Bundang Hospital, Seongnam, Republic of Korea; ^3^Department of Psychiatry, Seoul National University Hospital, Seoul, Republic of Korea; ^4^Department of Child Adolescent Psychiatry, Seoul National University College of Medicine, Seoul, Republic of Korea; ^5^Sensory Organ Research Institute, Seoul National University Medical Research Center, Seoul, Republic of Korea

## Abstract

*Objectives*. This study assessed the correlation between performance intelligence and the postoperative cochlear implant (CI) outcome in Korean-speaking children. In addition, the relationship between the performance intelligence subscales and the post-CI speech outcome was evaluated. *Materials and Methods*. Thirteen pediatric CI users (five males, eight females; median age at implantation 6.2 (range 1.3–14.2) years; median age at intelligence test 9.3 (range 5–16) years) who were tested using the Korean Educational Development Institute-Wechsler Intelligence Scale for children were studied. The correlations between the intelligence scores and 1-2 years postoperative Categories of Auditory Performance (CAP) scores and between subscales of performance and 1-2 years postoperative CAP scores were analyzed. *Results*. There was no correlation between the categories of verbal intelligence quotient (IQ) and performance IQ for “mentally retarded” and “average,” respectively (Spearman's rho = 0.42, *P* = 0.15). There was a strong correlation between performance IQ and the postoperative CAP scale (Spearman's rho = 0.8977, *P* = 0.0008). “Picture arrangement” and “picture completion,” reflecting social cognition, were strongly correlated with the postoperative CAP scales. *Conclusion*. Performance intelligence, especially social cognition, was strongly related to the postoperative CI outcome of cochlear implant users. Therefore, auditory rehabilitation, including social rehabilitation, should maximize the postoperative CI outcomes.

## 1. Introduction

Cochlear implant (CI) is a standard treatment option for children with profound hearing loss. However, the outcome of CI varies over a wide range among pediatric patients. Some prelingually deafened children show outstanding behavioral performance, such as the rapid acquisition of spoken language and the production of intelligible speech after years of CI-assisted rehabilitative effort, while other children develop awareness of environmental or speech sounds but never catch up with normal age-appropriate auditory language [1].

Therefore, it is relevant, both scientifically and clinically, to unravel the factors underlying the wide variability in CI outcome. Researchers have repeatedly suggested that demographic factors—such as age at the onset of severe-to-profound hearing loss, the duration of the severe-to-profound hearing loss, age at CI, and absence or presence of linguistic experience—are factors underlying the CI outcome variability [2–4]. Others have argued that the preoperative resting-state or task-driven cortical activity is a crucial indicator of an accurate individual prognosis [5–8].

In addition to these factors, recent studies have emphasized the role of the cognitive function of the subjects on the CI outcome [9]. Cognitive function tests consist of verbal and performance (nonverbal) tests. Since the feasibility of verbal testing is limited in deaf children, a performance test that presents tasks visually is important when evaluating the cognitive function of deaf subjects [10, 11]. Concerning the relationship between cognitive function and CI outcome, a study of Mandarin-speaking children using CI reported that the verbal intelligence quotient (IQ) might not represent the true intelligence of CI users [7]. However, there is little information on the correlation between the performance IQ subscales and postoperative CI outcome.

Therefore, we assessed the correlation between the performance IQ and postoperative CI outcome in prelingually deafened pediatric CI users. We also determined the performance IQ subscales that are most relevant to the CI outcome in Korean-speaking CI users using the Korean Educational Development Institute-Wechsler Intelligence Scale for Children (KEDI-WISC) [12].

## 2. Materials and Methods

### 2.1. Subjects

Of the children who underwent CI at the Department of Otorhinolaryngology-Head and Neck Surgery, Seoul National University Children's Hospital, 18 prelingually deafened children who were subjected to the KEDI-WISC to evaluate their verbal and performance IQs between 16 April 2009 and 5 August 2013 were initially included in this study. Children who had severe inner ear anomalies, cochlear nerve aplasia/hypoplasia, or severe bony cochlear nerve canal narrowing on temporal bone computed tomography (CT) or internal auditory canal magnetic resonance imaging (MRI) were excluded. Of the 18 initially included children, 5 with full-scale IQs classified as “severe mental retardation (full-scale IQ < 40)” were excluded because their verbal and performance IQ scores were unreliable. This study was approved by the Institutional Review Board of Seoul National University Hospital (number 1409-088-609).

The 13 children ultimately included comprised five males and eight females. Their median ages at CI and the KEDI-WISC test were 6.2 (range 1.3–14.2) and 9.3 (range 5–16) years, respectively. All of the children had profound sensorineural hearing loss (>90 dB HL on pure-tone audiometry or auditory brainstem response) in both ears. Depending on the age and compliance of the subject, the auditory brain stem response or auditory steady-state response were evaluated, or play audiometry or visual reinforcement audiometry was performed. The demographic characteristics of the included children are summarized in Table 1.

### 2.2. Korean Educational Development Institute-Wechsler Intelligence Scale for Children

We administered the KEDI-WISC to assess the intellectual function of the 13 children. The KEDI-WISC, a modified version of the Wechsler Intelligence Scale for children, is an intelligence test for Korean-speaking children between the ages of 5 and 15 years [13, 14]. This test consists of two subsets: verbal IQ and performance IQ [13, 14]. Both the verbal and performance IQ parts of the test were administered to all study participants using a standardized procedure by two pediatric clinical neuropsychologists, each with more than 10 years of clinical experience. The instructions were given to all children in a loud voice, and most children were able to understand the task through their hearing aids or CI. The examiner demonstrated each task to ensure that the children understood the instructions.

The verbal IQ test evaluates “information,” “similarities,” “arithmetic,” “vocabulary,” and “comprehension,” while the performance IQ test evaluates “picture completion,” “picture arrangement,” “block design,” “object assembly,” and “coding” (Table 2). Of the performance IQ subscales, “picture completion” and “picture arrangement” represent social cognition [15, 16], while “block design,” “object assembly,” and “coding” represent visual motor coordination [16, 17]. The total verbal and performance scores are obtained and can then be converted into the verbal and performance IQs by comparison with normative data for the general population of the same age. The full-scale IQ can be obtained by combining the verbal and performance IQs.

Using the verbal and performance IQ, intelligence is divided into seven categories: mentally retarded (IQ ≤ 69), borderline (70–79), low average (80–89), average (90–109), high average (110–119), superior (120–129), and very superior (≥130). Of note, an IQ ≤ 40 is categorized as “severely mentally retarded.” An IQ of 80 separates the intelligence categories into borderline and low average (Table 3).

### 2.3. Post-CI Outcome

The post-CI outcome was measured using the postoperative Categories of Auditory Performance (CAP) scale 1-2 (median, 1) years postoperatively. The CAP scores indicate the following: CAP 0 (no awareness of environmental sounds), 1 (awareness of environmental sounds), 2 (response to speech sounds), 3 (identification of environmental sounds), 4 (discrimination of some speech sounds without lip-reading), 5 (understanding of common phrases without lip-reading), 6 (understanding of conversation without lip-reading), and 7 (use of a telephone with a known listener) [19].

### 2.4. Analysis of Possible Related Factors

To compare the scores of the five performance IQ subscales, the Kruskal-Wallis test was performed. The Mann-Whitney* U*-test was used to compare pairs of subscales. Spearman's rank correlation test was performed to examine the correlations between the verbal and performance IQs, between the performance IQ and postoperative CAP scores, and between each performance subscale and the postoperative CAP scores. Statistical significance was set at *P* < 0.05. All analyses were performed using the Statistical Package for the Social Sciences (SPSS) 21.0 (SPSS, Chicago, IL).

## 3. Results

### 3.1. Verbal, Performance, and Full-Scale IQ

The mean full-scale IQ of the 13 subjects was 74.5 ± 19.0 (range 47–113), falling in the category “borderline.” When the full-scale IQ was subdivided into verbal and performance IQs, the mean verbal and performance IQs were 65.2 ± 21.2 (range 48–111) and 91.9 ± 17.5 (range 55–118), respectively. The categories of verbal and performance IQ were in the “mentally retarded” and “average” categories, respectively.

### 3.2. Subset Scores of the Performance IQ Test

**Figure 1:**
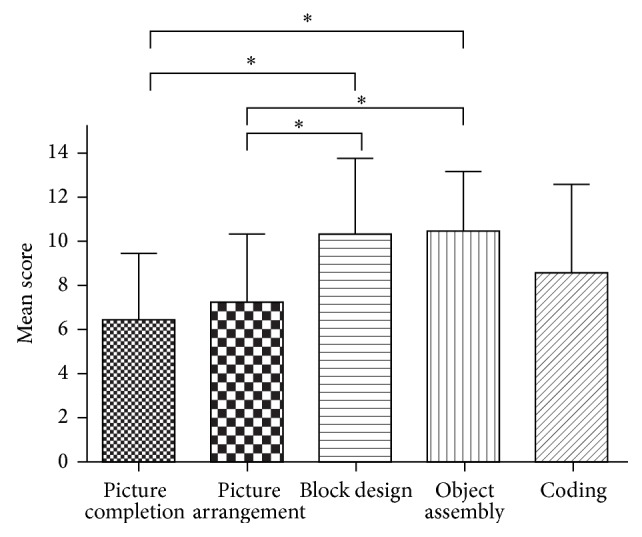
The mean (±SD) subscale scores for the performance IQ.

The mean scores for the five subsets of the performance IQ test are shown in Figure 1. There was a significant difference among the scaled scores for the five performance IQ subscales (*P* = 0.010, Kruskal-Wallis test).* Post hoc* individual Mann-Whitney* U*-tests revealed significant differences between “picture completion” and “block design” (*P* = 0.009), “picture completion” and “object assembly” (*P* = 0.002), “picture arrangement” and “block design” (*P* = 0.043), and “picture arrangement” and “object assembly” (*P* = 0.018). That is, the scores of the “picture completion” and “picture arrangement” subscales, reflecting social cognition, were significantly lower than those of “block design” and “object assembly,” reflecting visual motor coordination.

### 3.3. Correlation between Verbal and Performance IQs

**Figure 2:**
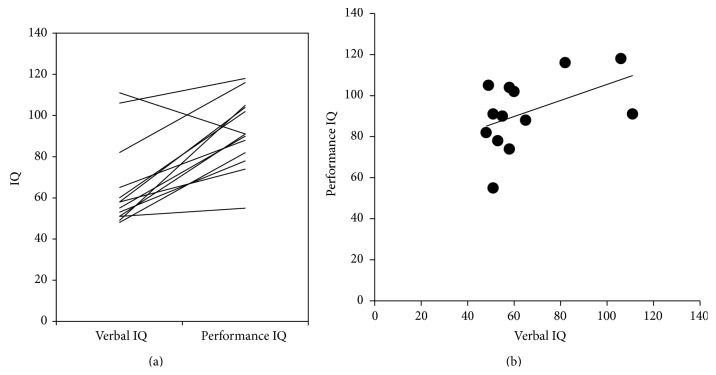
The raw data (a) and correlation (b) between the verbal and performance IQs. There was no correlation between the verbal and performance IQs (Spearman's rho = 0.4207, *P* = 0.1523).

Figure 2 shows the verbal and performance IQ data for each subject. The performance IQ was higher than the verbal IQ in all children, with a single exception (Figure 2(a)). There was no significant correlation between the verbal and performance IQs (Spearman's rho = 0.42, *P* = 0.15) (Figure 2(b)).

### 3.4. Correlation between Performance IQ and Postoperative CAP Score

**Figure 3:**
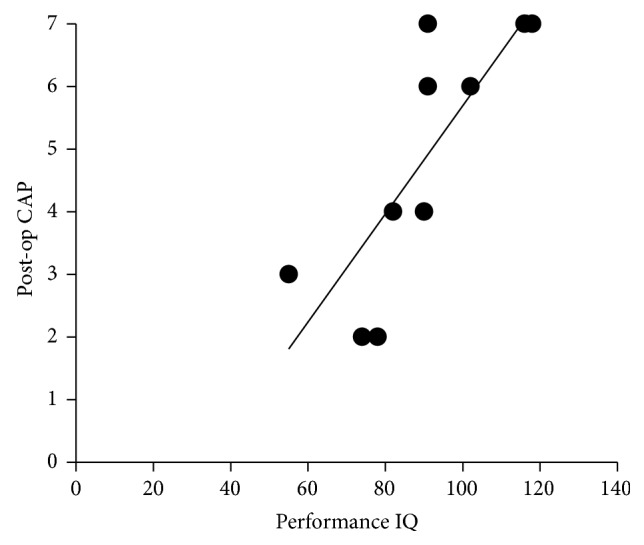
The correlation between the performance IQ and postoperative CAP score. There was correlation between the performance IQ and postoperative CAP score (Spearman's rho = 0.8977, *P* = 0.0008).

Postoperative 1-2 year CAP scores were available for 10 of the 13 children. There was a strong correlation between the performance IQ and postoperative CAP scores (Spearman's rho = 0.8977, *P* = 0.0008) (Figure 3).

### 3.5. Correlation between Each Subset of the Performance IQ and Postoperative CAP Scores

**Figure 4:**
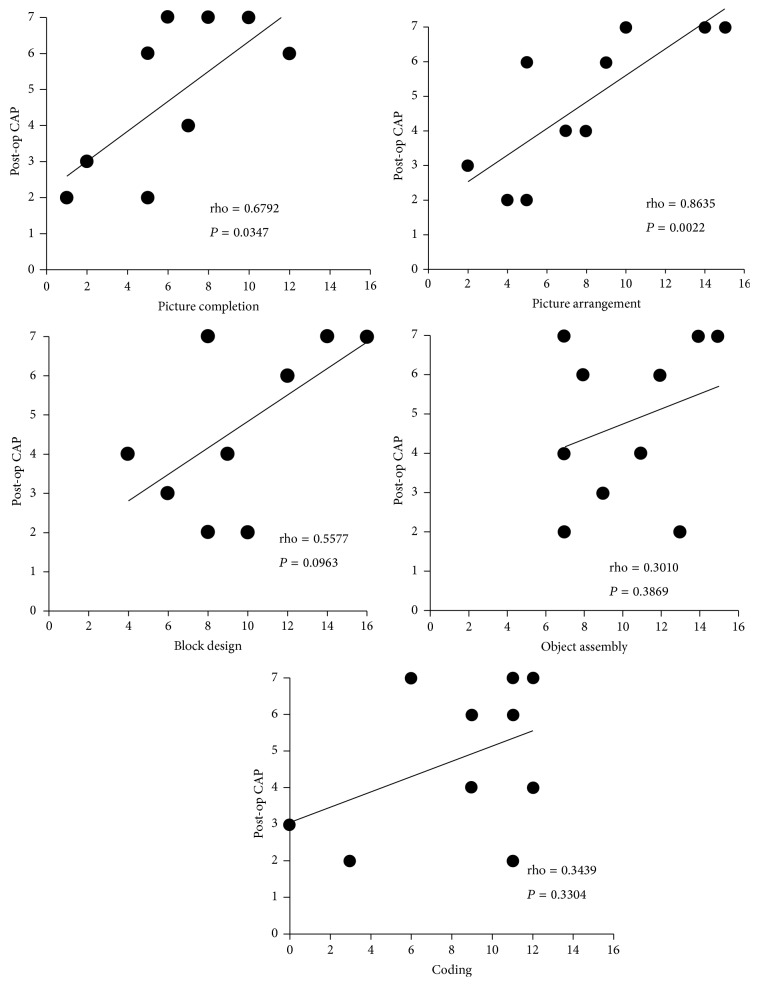
The correlation between each performance IQ subscale and the postoperative CAP score. “Picture arrangement” and “picture completion” had moderate to strong correlations with the postoperative CAP scores.

We evaluated the correlation between each subset of the performance IQ and the postoperative CAP scores. The “picture arrangement” subset had the highest correlation with the postoperative CAP scores, followed by “picture completion.” As mentioned above, “picture completion” and “picture arrangement” reflect social cognition. Therefore, we inferred that social cognition correlates well with the postoperative CAP scores (Figure 4).

## 4. Discussion

To our knowledge, this is the first study to assess the verbal and performance IQs of Korean-speaking CI users using the KEDI-WISC test. The mean performance IQ of our subjects fell in the category “average,” which is in agreement with previous reports [20–22].

There were discrepancies between the verbal and performance IQs in our CI subjects. Profiles between the verbal and performance IQs are often found in children with developmental disorders, such as autism (verbal IQ > performance IQ) [23–26], and in children with hearing loss (verbal IQ < performance IQ) [21]. In addition, our results showed that the postoperative CI outcome is linked to cognitive function, especially to performance IQ rather than verbal IQ. A recent study has indicated that better spoken language and verbal reasoning skills are correlated with the verbal IQ [27]. In this regard, our results may partially be attributed to the fact that the highest CAP score is not complex enough to assess auditory functions that are needed for development of the higher verbal reasoning skills that are tested in verbal IQ tests. Third, of the performance IQ subscales, “picture completion” and “picture arrangement,” which reflect social cognition, were associated with the post-CI outcome. This implies that not only intelligence but also social adaptation contributes to auditory rehabilitation after CI.

Verbal IQ reflects crystallized intelligence or knowledge coming from prior learning and past experiences [28]. Situations that require crystallized intelligence include reading comprehension and vocabulary examinations. This type of intelligence is based on facts and is rooted in experiences [28]. As we grow older and accumulate new knowledge and understanding, crystallized intelligence becomes stronger. Since deaf subjects do not go through these processes during the period of auditory deprivation before CI, their verbal IQ is lower. Conversely, performance IQ reflects fluid intelligence, which is the ability to perceive relationships independent of previous specific practices or instructions concerning those relationships. Therefore, the performance IQ of the CI subjects was comparable to that of their normal-hearing peers. This is in line with many previous reports [20–22].

Consistent with other research, we found that social cognition, as measured by “picture completion” and “picture arrangement,” is relatively poor in CI users [29]. In addition, our finding of relatively good visual-motor coordination measured using “block design” or “object assembly” in CI subjects is consistent with one previous report [30]. This has been attributed to brain plasticity; that is, visual-motor integration ability is improved secondary to auditory deprivation. Notably, the mean score of “coding” was poor, although it reflects the visual motor coordination ability. This can be explained by the fact that “coding” has the greatest relevance to verbal intelligence among the performance IQ subscales [31, 32]. In addition, “coding” is the only performance IQ subscale that uses letters, numbers, and symbols [33].

Social cognition focuses on how people process, store, and apply information about other people and social situations [34]. It focuses on the role played by cognitive processes in our social interactions. Social competence is closely related to social cognition. In CI users, some studies have reported that language skills are not related to social competence [35], while others reported a strong positive correlation between language skills and social competence [36]. We support the latter, and this suggests that postoperative CI outcomes are associated with social rehabilitation.

In this study, the postoperative CI outcomes were assessed using the CAP score. Many studies have evaluated CI outcomes using open-set phonetically balanced word recognition tests or sentence tests [9]. These tests can evaluate the auditory performance quantitatively, but they can be applied only to children older than 5 years. Since the CAP score can be easily applied and followed longitudinally over time, the improvement in the speech perception of each subject can be evaluated. Moreover, it is applicable to all subjects regardless of their intelligence, age, and other characteristics.

Limitations of this study must be mentioned. First, because this study presents preliminary results obtained from a small sample, a future study of a larger group of CI subjects is mandatory to draw generalized conclusions. Second, as the developmental stages of the subjects differed, a longer follow-up and longitudinal evaluation are needed. Third, a more reliable result might be achieved if patients with the same total performance IQ were included to compare the performance IQ subscales. Finally, to explore the causal relationship between the performance IQ and CI speech outcome, a prospective study that includes a preoperative performance IQ evaluation should be conducted.

## 5. Conclusion

Performance intelligence, especially social cognition, is correlated with the postoperative speech outcome in CI users. Therefore, postoperative rehabilitation—including a social rehabilitation program—might help to maximize the postoperative CI outcome.

## Figures and Tables

**Table 1:** Demographic characteristics of the included patients.

Male/female	5 : 8
Side of implantation, R/L	9 : 4
Age at implantation (median)	1.3 years to 14.2 years (6.2 years)
Age at intelligence test (median)	5 years to 16 years (9.3 years)
Bilateral profound sensorineural hearing loss	13 (100%)
Etiology of deafness	
Unknown (no inner ear anomaly)	11 (84.6%)
Inner ear anomaly (Mondini malformation, EVAS)	2 (15.4%)
Linguistic, pre-/postlingual	13 : 0

**Table 2:** The subsets of Korean Educational Development Institute-Wechsler.

Verbal IQ	
Information	A consecutive of orally presented questions that tap the child's general knowledge.
Similarities	A consecutive of orally presented of questions that ask how two words are alike or similar.
Arithmetic	A consecutive of arithmetic questions which the child solves mentally and gives answers.
Vocabulary	A consecutive of requirements that the child is asked to define a provided word.
Comprehension	A consecutive of questions about social situations or common concepts.

Performance IQ	

Picture completion	A series of pictures with a missing part, and the child is asked to identify the missing part by pointing and/or naming.
Picture arrangement	A series of pictures presented in an incorrect order, and the child is asked to place in the correct order to tell a story that makes sense.
Block design	A series of printed geometric pattern, and the child is asked to duplicate using red-and-white blocks.
Object assembly	A series of fragments of common objects, each presented in a standardized shape, and the child is asked to assemble to form a meaningful whole.
Coding	A series of simple shapes, each paired with a code. The child asked to draw the shape in its corresponding code.

**Table 3:** The diagnostic categories of intelligence quotient.

Category	Scaled score	IQ
Very superior	≥13	≥130

Superior	12	120–129

High average	11	110–119

Average	10	90–109
	9	

Low average	8	80–89

Borderline	7	70–79

Mental retardation	6	55–69: mild
	5	40–55: moderate
	<4	<40: severe

Adopted form [18].
